# Evolution of neural computations: Mantis shrimp and human color decoding

**DOI:** 10.1068/i0662sas

**Published:** 2014-09-17

**Authors:** Qasim Zaidi, Justin Marshall, Hanne Thoen, Bevil R. Conway

**Affiliations:** Graduate Center for Vision Research, State University of New York, New York; e-mail: qz@sunyopt.edu; Queensland Brain Institute, The University of Queensland, Brisbane, Queensland 4072, Australia; e-mail: h.thoen@uq.edu.au; Queensland Brain Institute, The University of Queensland, Brisbane, Queensland 4072, Australia; e-mail: justin.marshall@uq.edu.au; Neuroscience Program, Wellesley College, Wellesley, Massachusetts; e-mail: bconway@wellesley.edu

**Keywords:** mantis shrimp, primate color vision, color decoding, tuning curves, winner-take-all, photoreceptors, IT cortex

## Abstract

Mantis shrimp and primates both possess good color vision, but the neural implementation in the two species is very different, a reflection of the largely unrelated evolutionary lineages of these creatures. Mantis shrimp have scanning compound eyes with 12 classes of photoreceptors, and have evolved a system to decode color information at the front-end of the sensory stream. Primates have image-focusing eyes with three classes of cones, and decode color further along the visual-processing hierarchy. Despite these differences, we report a fascinating parallel between the computational strategies at the color-decoding stage in the brains of stomatopods and primates. Both species appear to use narrowly tuned cells that support interval decoding color identification.

In the second half of the 19th century, James Clerk Maxwell showed that people make color matches by equating light absorbed in each of three photoreceptor classes. Maxwell's results supported the idea that color is represented in the human brain by a linear three-dimensional (3-D) space in which distinct points correspond to different colors, while each point (color) within this space corresponds to an almost infinite number of physically distinct lights (metamers). For example, the single-wavelength yellow of the rainbow is indistinguishable from an appropriate mixture of wavelengths that separately appear red and green—both stimuli cause the same relative activation of the three cone types. Maxwell's discovery pointed to the critical role that neural comparison of photoreceptor outputs plays in determining what colors we see.

When Cronin and Marshall (1989) reported that mantis shrimp, a predatory stomatopod crustacean, has 12 classes of narrowly tuned photoreceptors (Figure 1A), three in the ultra-violet range and nine covering the 400–700-nm spectrum, the scientific imagination ran wild: do they have a 12-dimensional (12-D) color space, so that they distinguish colors we confuse, and see colors we cannot even imagine? Such conjectures were restrained by the concern that their small brains could be overloaded by color computations in a 12-D space. Behavioral experiments by Thoen, How, Chiou, and Marshall (2014) have since shown that mantis shrimp are in fact poor at discriminating colors that humans see as distinct. The results suggested that the 12 classes of photoreceptors function independently, and their outputs are not compared by later neurons. So it has been concluded that mantis shrimp have a color system unlike humans, or possibly any other creature. The requirements of rapid hunting decisions and a small brain, could have led mantis shrimp to evolve 12 narrow-tuned color receptors at the front end of the visual system: presumably the photoreceptors feed a fast, hard-wired, interval-decoding computation, where perceived color corresponds to the peak sensitivity of the most responsive photoreceptor. Such hard-wiring is typical of many invertebrate sensory systems where behavioral tasks are “matched” to the environmental parameters that drive the task.

**Figure 1.**
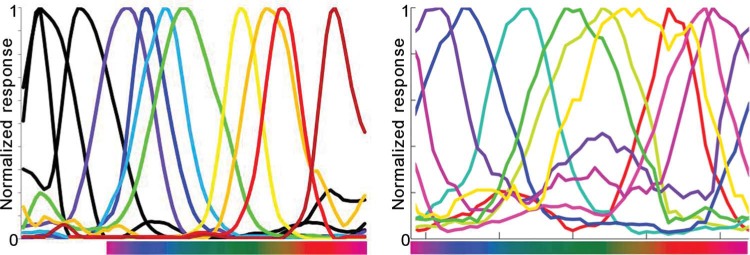
Color tuning of (A) mantis shrimp photoreceptors, and (B) of a few neurons in macaque inferior temporal cortex.

The eyes and photoreceptors of mantis shrimp and humans are clearly different, but are the neural strategies used to compute color that different? On the basis of physiological and anatomical research in macaque monkeys, a trichromat with color vision very similar to humans (Stoughton, Lafer-Sousa, Gagin, & Conway, 2012), we have reason to believe that the computations carried out by the color-vision systems in humans and mantis shrimp are more similar than they first appear. Although color in trichromatic primates is encoded using three (not 12) classes of broadly tuned photoreceptors, primates have much larger brains than shrimp: neural circuits compare cone responses within the retina (Sun, Smithson, Zaidi, & Lee 2006), and the neural circuits responsible for color perception are linked across several different cortical regions (Conway, 2014). In inferior temporal cortex (IT), several steps downstream of the cones, the cells are remarkably color specific (Komatsu, Ideura, Kaji, & Yamane, 1992), as shown for a sample of IT neurons in Figure 1B (Conway, Moeller, & Tsao, 2007). Some cells respond only to red, others to reddish blue, bluish red, violet, and so on. In their specificity, the color preferences of these cells are strikingly similar to the color specificity of the mantis shrimp photoreceptors, suggesting that the 400 million year old color processing system in stomatopods and the 40 million year old primate system could ultimately use a similar strategy at the decoding stage.

To test this idea, we used simulations to determine the extent to which primates could use narrowly tuned IT cells for an interval-color-decoding strategy similar to the one that is postulated to operate in the mantis shrimp. The strategy hypothesizes that the decoded color of a stimulus corresponds to the color preference of the IT neuron that produced the highest firing to the stimulus. In formal terms, this approach couples interval coding with a winner-take-all decision rule. For each of 279 posterior IT “glob” cells, based on responses to brief presentations of 45 colors measured with single-unit recording (Conway et al., 2007), we simulated a model cell with the same color-tuning. Firing rates for each stimulus color were generated by a Poisson distribution with mean and variance equal to the mean firing rate of the measured cell. So in every trial, the simulated response varied around the color-tuning by an amount chosen at random from the Poisson distribution. Each frame of the movie in Figure 2 (left—movie can be found at http://i-perception.perceptionweb.com/journal/I/volume/5/article/i0662sas) shows the simulated responses of the whole population to each color stimulus. The cells have been sorted along the *x*-axis according to their color preferences: cells tuned to red are on the left, followed rightwards by cells tuned to orange, yellow and so on around the color circle. The stimulus is depicted by the red symbol, and the decoded color is simply the color preference of the cell with the maximum firing rate. For the first trial, the cell with the maximum firing is tuned to the stimulus color, showing that the simple decoding strategy worked. As the simulated stimulus changes from 1 to 45, even with this meager number of cells and stimuli, the population supports fairly accurate interval decoding of color. Since each cortical neuron receives thousands of synapses, and color cells are organized into local columns of similarly color-tuned cells, it may be unrealistic to restrict the decision to a single neuron's response. So we used the average of the responses of all cells with the same preferred color, and found that the decoding accuracy improved markedly (Figure 2—right). The success of interval decoding presents a physiologically realistic and computationally efficient alternative to color theories based on unique hues (De Valois & De Valois, 1993) that have no physiological support. Interval decoding is also compatible with the results of the only color micro-stimulation experiment done on humans (Murphey, Yoshor, & Beauchamp, 2008).

**Figure 2.**
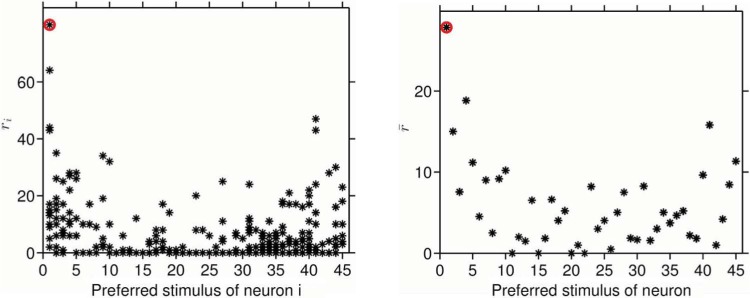
Stills of movies that can be found at http://i-perception.perceptionweb.com/journal/I/volume/5/article/i0662sas. (Left) In the movie each frame shows Poisson responses of 279 IT neurons (black stars) elicited by a stimulus color (red circle) on one trial, plotted versus the mode of the tuning curve of each cell. The stimulus color progresses on each frame representing an independent trial. At the end of the 45-color cycle, blue circles plot the decoded color (the preferred color of the cell that fired maximally on that trial) against the stimulus color. The simulations are repeated five times to demonstrate the variability in probabilistic decoding. (Right) In the movie the black stars now give the average response of all the cells preferentially tuned to the color on the *x*-axis. The decoding is considerably more accurate.

It is intriguing to consider whether winner-take-all with IT cells represents a hard-wired approximation of optimal Bayesian decoding of the population of responses. If the neurons in the population have independent variability, then the population response probability will be the product of the Poisson probability of all the neurons. Applying Bayes' rule to get the probability of decoding a stimulus color given a pattern of population responses, leads to an expression for decoding that contains a term that represents multiplication of tuning curves raised by the number of spikes, and is the only term that depends on the pattern of responses on a trial. A cell that only fires if it gets spikes from two cells within a short time interval, will only fire for stimuli for which the tuning curves of the earlier cells overlap, i.e. the output tuning curve will look like a multiplication of the input tuning curves. Similarly, a cell that only fires if it receives a defined number of input spikes in a short interval, will have a tuning curve that looks like the input tuning curve raised to the power of the number of spikes (Sanger, 1998). These operations will approximate the response-dependent term in Bayesian decoding, and performed on broadly tuned outputs from antecedent stages of processing will generate narrower tuning, consistent with empirical observations in IT. Interval decoding would therefore provide rapid color identification because no further computations would be required on the outputs of IT neurons. Since IT cortex has millions of color-tuned cells, they can sample the spectrum much more finely than the 12 mantis shrimp photoreceptors, so interval decoding could simultaneously provide much better color identification and discrimination compared to mantis shrimp, resolving the paradox that mantis shrimp have poorer color vision than humans despite having more photoreceptor classes.

In mantis shrimp, the cost of early functional specialization in the compound eye, and the sub-division of tasks to different eye areas (Cronin & Marshall, 1989), requires that the animal scan the scene to generate a representation of its visual world (Land, Marshall, Brownless, & Cronin, 1990). The primate eye is fundamentally different from the shrimp, like a digital camera it possesses a single focusing apparatus for a dense array of photoreceptors. Using just three classes of broadly tuned cone photoreceptors, the primate visual system is able to distinguish the spectra of natural lights and objects sufficiently (Barlow, 1982), while maintaining good spatial resolution, and providing the means to identify objects by their colors despite variations in ambient lights and surrounding scenes (Zaidi, 1998, 2001). More classes of photoreceptors would improve the sampling of natural spectra (Nascimento, Foster, & Amano, 2005), but would seriously compromise spatial resolution. Generating narrow color tuning in functionally specialized cortical regions affords rapid interval decoding without losing these features.

Despite tremendous differences in human versus mantis shrimp eye structure and brain circuitry, the striking similarity between the color sensitivities of primate IT neurons and stomatopod photoreceptors provides evidence of a common computational strategy across largely unrelated species. Interval decoding of color is an interesting example of independent evolutionary histories converging on the same robust computational principle, and may thus be worth emulating by machine vision systems designed to function in the real word.
